# Hepatotoxicity in immune checkpoint inhibitors: A pharmacovigilance study from 2014–2021

**DOI:** 10.1371/journal.pone.0281983

**Published:** 2023-03-07

**Authors:** Ze Xu, Guanpeng Qi, Xin Liu, Zhaohang Li, Aijun Zhang, Juman Ma, Zuojing Li

**Affiliations:** Department of Pharmaceutical informatics, School of Shenyang Pharmaceutical University, Shenyang, Liaoning, China; UT MD Anderson Cancer Center, UNITED STATES

## Abstract

Adverse events(AEs) related to hepatotoxicity have been reported in patients treated with immune checkpoint inhibitors (ICIs). As the number of adverse events increases, it is necessary to assess the differences in each immune checkpoint inhibitor regimen. The purpose of this study was to examine the relationship between ICIs and hepatotoxicity in a scientific and systematic manner. Data were obtained from the FDA Adverse Event Reporting System database (FAERS) and included data from the first quarter of 2014 to the fourth quarter of 2021. Disproportionality analysis assessed the association between drugs and adverse reactions based on the reporting odds ratio (ROR) and information components (IC). 9,806 liver adverse events were reported in the FAERS database. A strong signal was detected in older patients (≥65 years) associated with ICIs. hepatic adverse events were most frequently reported with Nivolumab (36.17%). Abnormal liver function, hepatitis, and autoimmune hepatitis were most frequently reported, and hepatitis and immune-mediated hepatitis signals were generated in all regimens. In clinical use, patients should be alert to these adverse effects, especially in elderly patients, who may be aggravated by the use of ICI.

## 1. Introduction

Immune checkpoint inhibitors (ICIs) are a new class of cancer therapeutics that include programmed death receptor 1 monoclonal antibodies (nivolumab, cemiplimab, pembrolizumab), cellular programmed death ligand 1 monoclonal antibodies (atezolizumab, avelumab, durvalumab) and Cytotoxic T-lymphocyte-associated protein 4 monoclonal antibodies (ipilimumab, tremelimumab) [1]. PD-1 and PD-L1 monoclonal antibodies reactivate suppressed T cells by blocking the interaction between PD-1 and PD-L1, allowing them to exert their original immune action and kill tumor cells. In contrast, CTLA-4 class monoclonal antibodies maintain T cell activation by disabling signals that inhibit T cell activation [2–4].

In recent studies, ICIs have shown significant efficacy, changing the outlook for the treatment of non-small cell lung cancer(NSCLC) [5], squamous cell carcinoma [6], hepatocellular carcinoma [7], renal cell carcinoma [8] and uroepithelial carcinoma [9]. In phase 3 studies in unresectable hepatocellular carcinoma, atezolizumab was the first ICIs to demonstrate improved overall survival and progression-free survival. In addition to ICIs monotherapy, a combination of CTLA-4 and PD-1 inhibitors has been approved for the treatment of certain malignancies. However, due to their specific mechanism of action, ICIs may lead to disruption of the patient’s normal body systems and adverse drug reactions (ADRs) while improving antitumor efficacy. Any organ system can be affected by immune-related adverse events (irAEs), but the most common organs are the skin, liver, gastrointestinal tract, lungs and endocrine glands [10]. The utility of ICI therapy is limited by irAEs in multiple organ systems. Some studies have shown that the probability of ICI-related hepatotoxic adverse events(AEs) is 2% and clinically significant ICI-related hepatotoxicity is uncommon, but most hepatotoxic AEs can lead to permanent discontinuation of the drug [11]. The indications for ICI continue to expand into different cancers, stages of disease. With this increased use, adverse events including immune checkpoint inhibitor-related hepatotoxicity have become an important clinical issue.

FAERS is a spontaneous reporting system that includes all adverse drug reactions and medication error information collected by the US Food and Drug Administration (FDA) and provides an important basis for post-marketing safety risk monitoring and evaluation of drugs. There is no safety analysis of ICIs-related hepatotoxicity in a large data sample. Therefore, this study provides a reference for rational clinical use of drugs by data mining and analysis of safety signals of ICIs in the FAERS database, the adverse event reporting system of the FDA.

## 2. Methods

### 2.1 Data

Data for the retrospective pharmacovigilance study were obtained from the FAERS database. FAERS collects data not only from the United States, but also from other countries and regions. A total of 36 quarterly reporting files were screened from the first quarter of 2014 to the fourth quarter of 2021.FAERS data files contain seven types of data sets: patient demographic and administrative information (DEMO), drug/biologic information (DRUG), adverse events (REAC), patient outcomes (OUTC), reporting source (RPSR), reporting drug therapy start date and end date (THER) and indication (INDI). As recommended by the FDA, we removed duplicate records prior to statistical analysis by selecting the most recent EVENT_DT when the CASEID was the same and the higher PRIMARYID when the CASEID and EVENT_DT were the same. All data downloaded from the FDA website were processed by SAS 9.4 and further analyzed using R software.

### 2.2 Targeted drugs and AEs

We used the trade names and generic names of drugs included in the National Center for Biotechnology Information(NCBI) to search the FAERS database for ICIs that have been approved for marketing by the FDA, including CTLA-4 (ipilimumab, tremelimumab), PD-1 (nivolumab, cemiplimab, pembrolizumab) and PD-L1 (atezolizumab, avelumab, durvalumab) (S1 Table).

Adverse events with ICIs related to hepatotoxicity were defined as cases in the FAERS database where the treatment regimen included drugs in the ICIs class and a liver-related adverse reaction in the SOC classification occurred. AEs in the FAERS database are coded according to the preferred terms (PTs) in the Medical Dictionary of Regulatory Activities (MedDRA). According to MedDRA version 23.0, our study includes all liver and hepatobiliary-like diseases (MedDRA code 10019654) and all tumors of the hepatobiliary system (MedDRA code 10019811). In addition, based on the structure and variables of the FAERS database, a single adverse event report of ICIs related to hepatotoxicity was recorded as one case of data, even if more than one adverse event report was reported by the same patient.

### 2.3 Time interval analysis of the occurrence of ICI-related hepatotoxic AE

We assessed the time to occur of ICIs-related hepatotoxic AEs. The time of occurrence was the time interval between START_DT (treatment start date) and EVENT_DT (date of adverse event). We excluded inaccurate data entry, missing specific data and incorrectly entered reports (EVENT_DT earlier than START_DT). Reported hepatotoxic adverse events were used as positive endpoint events of interest, with the time of initiation of treatment in the case as the starting point of the event and the time of reporting of AEs as the positive endpoint of interest. Kaplan-Meier curves were used to calculate the median survival time and to describe the course of AE occurrence in terms of survival curves. The Kruskal-Wallis test was used to determine whether the time to AE onset was statistically different between ICI treatment regimens. A two-by-two comparison of each drug was then performed by Wilcoxon rank sum test.

### 2.4 Statistical analysis

#### 2.4.1 Disproportionality analysis

Disproportionality analysis is a data mining method that is now widely used in adverse drug reaction monitoring, and this method uses the number of times a drug is reported in association with an event in the adverse drug event reporting database as a basis to study the statistical relationship between the target drug and the target event in the database [12]. There are two main types of proportional imbalance methods applied nowadays: the frequency method and the Bayesian method, and the frequency method contains the reporting odds ratio method and the proportional reporting ratio method. The advantage of the frequency method is that it is simple to calculate, easy to understand, low time consuming, and does not require a priori information about the model, but it is highly susceptible to singular values. When the number of frequencies is small, the accuracy of the algorithm will be affected. The BCPNN method takes into account not only the information of probability asymmetry but also the information of the overall sample, which is more flexible and stable compared with the frequency method. In data scenarios with larger sample size, the frequency method has more aggressive calculation results, while the BCPNN method is relatively conservative. Therefore, in practical applications, the two methods should be combined to evaluate the signal results of pharmacovigilance in an integrated manner.

AE reports for suspected drugs and other drugs were calculated using a 2×2 columnar table (Table 1). Two data mining methods, reporting odds ratio (ROR) [13] and Bayesian confidence propagation neural network (BCPNN) [14] for information components (IC), were used to detect potential associations between ICI and liver AEs.

**Table 1:** Proportional imbalance analysis 2*2 columnar table.

	Reports with hepatotoxicity	Reports without hepatotoxicity
Reports with the suspected drugs	a	b
All other reports	c	d

#### 2.4.2 Reporting odds ratio method

The formula for calculating the ROR is as

$$ROR=\frac{a/c}{b/d}=\frac{ad}{bc}$$

The 95% confidence interval for the ROR can be calculated by

$$95\%CI=e^{\ln\left(ROR\right)\pm 1.96\sqrt{\left(\frac{1}{a}+\frac{1}{b}+\frac{1}{c}+\frac{1}{d}\right)}}$$

#### 2.4.3 BCPNN method

According to Bayes’ theorem, the calculation of IC can be obtained as

$$IC={log}_{2}\left[\frac{P(Drug|ADR)}{P(ADR)}\right]={log}_{2}\left[\frac{P(Drug,ADR)}{P(Drug)P(ADR)}\right]$$

Let *P(Drug)* obey a Beta distribution with α_1_, α_2_ as prior parameters, *P(ADR)* obey a Beta distribution with β_1_, β_2_ as prior parameters and *P(Drug*, *ADR)* obey a joint Beta distribution with γ_1_, γ_2_ as prior parameters. When the Bayesian prior probabilities are uninformative priors, the Beta distribution is represented as

$$P(Drug)∼Beta(\alpha_{1}=1,\alpha_{2}=1)$$

$$P(ADR)∼Beta(\beta_{1}=1,\beta_{2}=1)$$

$$P(Drug,ADR)∼Beta(\gamma_{1}=1,\gamma_{2})$$

where

$${\gamma \prime }_{2}=\frac{\gamma_{1}+a}{{P(Drug)}_{pos}{P(ADR)}_{pos}}-a-\gamma_{1}$$

The 95% confidence interval for IC was calculated by

$${IC}_{025}=E(IC)\pm 1.96\sqrt{V(IC)}$$

*E(IC)* and *V(IC)* can be expressed in a more understandable and computer-friendly way by

$$E(IC)=\frac{1}{ln2}[\Psi (a+\gamma_{1})-\Psi (a+\gamma_{1}+{\gamma^{\prime }}_{2})-\Psi (a+b+\alpha_{1})-\Psi (\alpha_{1}+\alpha_{2}+N)+\Psi (a+c+\beta_{1})-\Psi (\beta_{1}+\beta_{2}+N)]$$

$$V(IC)=\frac{1}{{ln}^{2}2}[\Psi \prime (a+\gamma_{1})-\Psi \prime (a+\gamma_{1}+{\gamma^{\prime }}_{2})-\Psi \prime (a+b+\alpha_{1})-\Psi \prime (\alpha_{1}+\alpha_{2}+N)+\Psi \prime (a+c+\beta_{1})-\Psi \prime (\beta_{1}+\beta_{2}+N)]$$

## 3. Results

### 3.1 Descriptive analysis

By processing, a total of 53,000,662 data were recorded in the FAERS database between 2014 and 2021, of which 269,090 ICI-related adverse events were reported, of which 9,806 were reported to be related to hepatotoxicity. We summarized the clinical characteristics of the patients, which are described in Table 2.

**Table 2:** Clinical characteristics of adverse events associated with ICIs.

	Hepatotoxicity AEs with ICIs(9,806)	Hepatotoxicity AEs with other drugs(592,461)	IC025	ROR025
**Sex group**				
Male	5653(57.65%)	305163(44.95%)	**0.0006**	**0.5721**
Female	3213(32.77%)	298808(43.75%)	**0.3431**	**1.6807**
Intersex	0(0.00%)	5(0.00%)		
Uuknowen	0(0.00%)	39(0.01%)		
Missing	940(9.59%)	75315(11.29%)		
**Age group(years)**				
≥65	3607(36.78%)	156055(26.34%)	**0.2617**	**1.3984**
<65	4364(44.50%)	276045(46.59%)	-0.2721	0.6542
MISS	1835(18.71%)	160361(27.07%)		
**Outcome**				
other serious	5028(51.27%)	321148(54.21%)	-0.1648	0.8377
hospitalization	3304(33.69%)	172836(29.17%)	**0.1101**	**1.1408**
death	1003(10.23%)	53328(9.00%)	**0.0492**	**1.0482**
life-threatening	298(3.04%)	18147(3.06%)	-0.2176	0.8597
disability	66(0.67%)	5128(0.87%)	-0.7503	0.5926
RI	9(0.09%)	364(0.06%)	-0.5358	0.7513
Congenital anomaly	2(0.02%)	707(0.12%)		
miss	96(0.98%)	20802(3.51%)		
**Reporting region(Top five hepatotoxicity AEs with ICIs)**				
Japan	2900(29.57%)	69396(11.71%)	1.2394	3.0286
America	1770(18.05%)	179364(30.27%)	-0.8098	0.4817
France	1289(13.15%)	87909(14.84%)	-0.2567	0.8188
Germany	554(5.65%)	26396(4.46%)	0.2098	1.1776
China	318(3.24%)	18386(3.10%)	-0.1024	0.9349

A greater proportion of all reports of hepatotoxicity associated with ICIs were in males than in females (57.65% vs. 32.77%). By further analysis, signals were detected in both males and females (Male: IC025 = 0.0006, ROR025 = 0.5721; Female: IC025 = 0.3431, ROR025 = 1.6807). According to the United Nations definition of the age of the elderly, developed countries define 65 years or older as the elderly, so 65 years was used as the cut-off for classification. The results of the study showed significant differences between age subgroups, with a smaller proportion of elderly (≥65) than non-elderly (<65) (36.78% vs. 44.50%), but further testing produced a significant signal (IC025 = 0.2617, ROR025 = 1.3984), which may be attributed to the effect of cancer survival and degenerative changes in the elderly organism. The most frequently reported regressions were other serious medical events, hospitalization and death. Hospitalizations (IC025 = 0.1101, ROR025 = 1.1408), deaths (IC025 = 0.0492, ROR025 = 1.0482) associated with liver AEs following ICI treatment were reported, indicating the threatening nature of potential life-ICIs-related hepatotoxicity. The most reported countries were Japan (29.57%), followed by the USA (18.05%), France (13.15%), Germany (5.65%) and China (3.24%).

In all reports of ICIs-related hepatotoxicity, we analyzed the relationship between each class of ICIs and age separately (Table 3). atezolizumab, Ipilimumab, Nivolumab and Pembrolizumab were detected as signals in the elderly population.

**Table 3:** The signals of age at ICIs-related hepatotoxic adverse events.

	Age	Count	IC025	ROR025
Atezolizumab	<65	470	-0.3854	0.5994
	≥65	390	**0.1457**	**1.2750**
Avelumab	<65	52	-0.6358	0.5249
	≥65	37	-0.3858	0.8198
Cemiplimab	<65	11	-	-
	≥65	516	-	-
Durvalumab	<65	143	**0.1281**	**1.7086**
	≥65	764	-1.0041	0.4040
Ipilimumab	<65	554	-0.2663	0.7032
	≥65	1445	**0.0670**	**1.1422**
Nivolumab	<65	1389	-0.4134	0.5482
	≥65	470	**0.3402**	**1.5735**
Pembrolizumab	<65	934	-0.5616	0.4549
	≥65	1067	**0.4467**	**1.8435**
Tremelimumab	<65	172	**0.1421**	**2.4948**
	≥65	26	-2.0469	0.1757

Differences in various specific hepatotoxic adverse events were observed in all ICIs regimens. Of all hepatotoxic adverse events reported, abnormal liver function (1191, 12.15%), hepatitis (1122, 11.44%), liver disease (843, 8.60%), autoimmune hepatitis (664, 6.77%), Drug-induced liver injury (606, 6.18%), immune-mediated hepatitis (590,6.02%) and ascites (521 5.31%) were the most commonly reported (Table 4). These reports accounted for 56.47% of all reports.

**Table 4:** Frequency of hepatotoxic AEs related to ICIs drugs.

Hepatotoxicity AEs	COUNT	PERCENT
Hepatic function abnormal	1191	12.15%
Hepatitis	1122	11.44%
Liver disorder	843	8.60%
Autoimmune hepatitis	664	6.77%
Drug-induced liver injury	606	6.18%
Immune-mediated hepatitis	590	6.02%
Ascites	521	5.31%
Others	4269	43.53%

### 3.2 Spectrum of hepatotoxic AEs in immunotherapy regimens

In general, not all ICIs were associated with hepatic AEs. However, signals were detected when each drug was analyzed separately with hepatotoxic AEs. Tremelimumab had the strongest statistical association with ICIs-associated hepatotoxic AEs in the analysis of overall and individual ICIs (Table 5).

**Table 5:** Signals for overall and each class of ICIs drugs with hepatotoxic AEs.

	a	b	c	d	IC025	ROR025
Atezolizumab	1070	21343	601197	52377052	**1.9773**	**4.1074**
Avelumab	106	3274	602161	52395121	**1.1558**	**2.3216**
Cemiplimab	78	1835	602149	52396560	**1.4616**	**2.9486**
Durvalumab	722	14151	601545	52384244	**1.9796**	**4.1228**
Ipilimumab	1631	41345	600636	52357050	**1.6658**	**3.2725**
Nivolumab	3547	107784	598720	52290611	**1.4380**	**2.7793**
Pembrolizumab	2444	67085	599823	52331310	**1.5693**	**3.0525**
Tremelimumab	208	2467	602059	52395928	**2.5277**	**6.3692**
Total	9806	259284	592461	52139111	**1.6515**	**3.2613**

(a) The number of records with liver AEs reported for ICIs. (b) The number of records with any other AEs reported for ICIs. (c)The number of records with any liver AEs for other drugs. (d) The number of records reported other AEs for other drugs. ROR,reporting odds ratio; ROR025, the lower end of the 95% confidence interval of ROR; IC, information component; IC025, the lower limit of the 95% confidence interval of IC.

**Fig 1:**
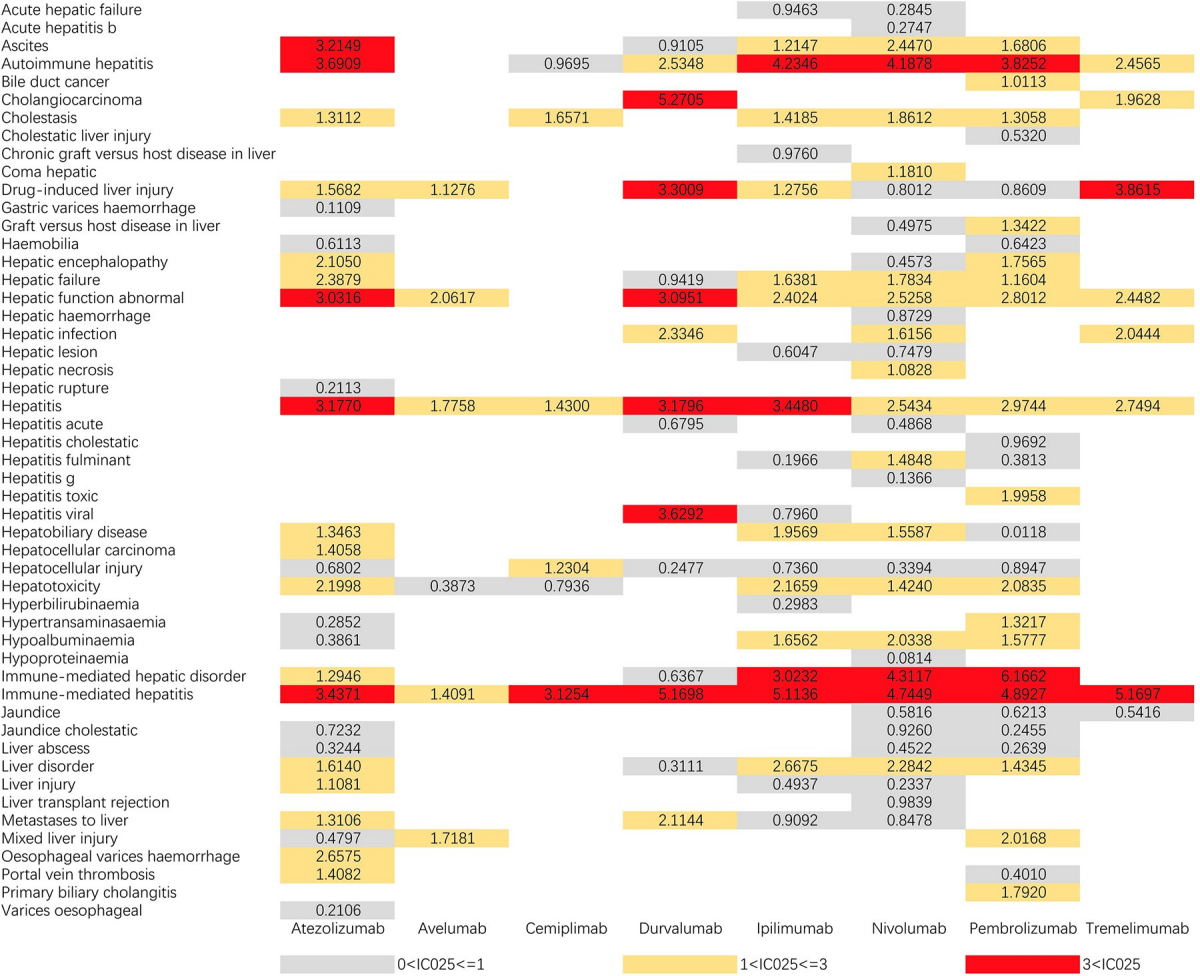
IC025 values for ICIs-related hepatotoxic adverse events. PT, preferred term; IC025, the lower end of the 95% confidence interval of IC.IC025 greater than 0 was deemed a signal.

**Fig 2:**
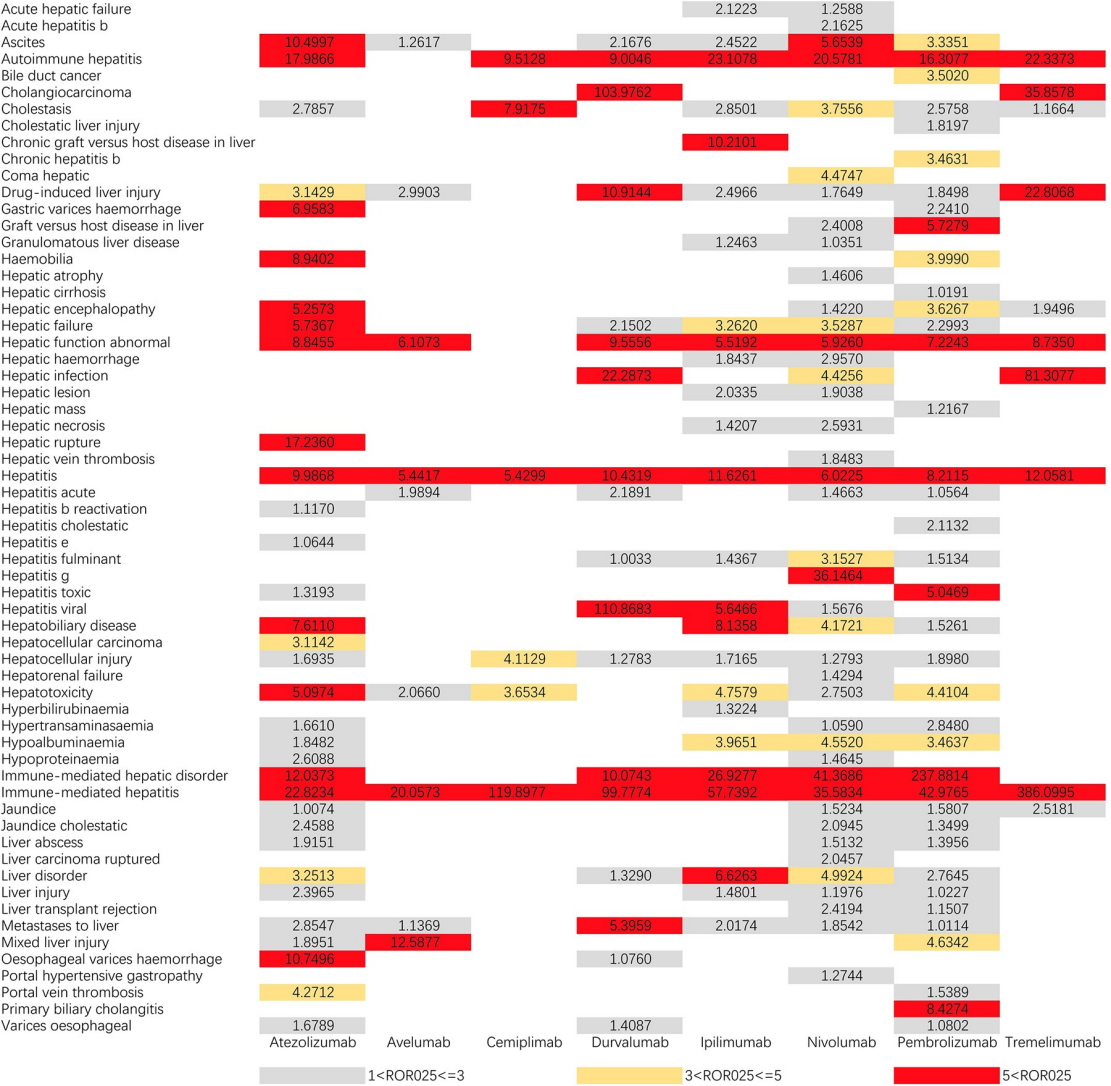
ROR025 values for ICIs-related hepatotoxic adverse events. PT, preferred term; ROR025, the lower end of the 95% confidence interval of ROR.ROR025 greater than 1 was deemed a signal.

Figs 1 and 2 demonstrate IC025 and ROR025 between PTs for drugs and adverse events, respectively. nivolumab had the widest range of hepatic AEs, with 33 PTs being monitored for signals ranging from liver injury (IC025 = 0.2237, ROR025 = 1.1976) to immune-mediated liver injury (IC025 = 4.3117, ROR025 = 41.3686). 28 PTs were found to be significantly associated with Atezolizumab treatment, ranging from oesophageal varices (IC025 = 0.2106, ROR025 = 1.6789) to immune-mediated hepatitis (IC025 = 3.4371, ROR025 = 22.8234). 29 PTs were found to be significantly associated with Tremelimumab had a significant association, encompassing from cholestatic jaundice (IC025 = 0.2106, ROR025 = 1.6789) to immune-mediated hepatitis (IC025 = 3.4371, ROR025 = 22.8234). 22 PTs were monitored as signals for Ipilimumab, ranging from fulminant hepatitis (IC025 = 0.1966, ROR025 = 1.4367) to immune-mediated liver injury (IC025 = 5.1136, ROR025 = 57.7392). durvalumab had 15 PTs monitored as signals ranging from hepatocellular injury (IC025 = 0.2477, ROR025 = 1.2783) to viral hepatitis (IC025 = 3.6292, ROR025 = 110.8683). 9 PTs were monitored for signal with Tremelimumab and 6 PTs were detected for each of Avelumab and Cemiplimab, with all three having the strongest association with immune-mediated hepatitis. Hepatitis and immune-mediated hepatitis were detected as signals across all drugs, with the widest distribution of hepatotoxic AEs in the ICI.

### 3.3 Analysis of the time interval of the occurrence of AEs

**Fig 3:**
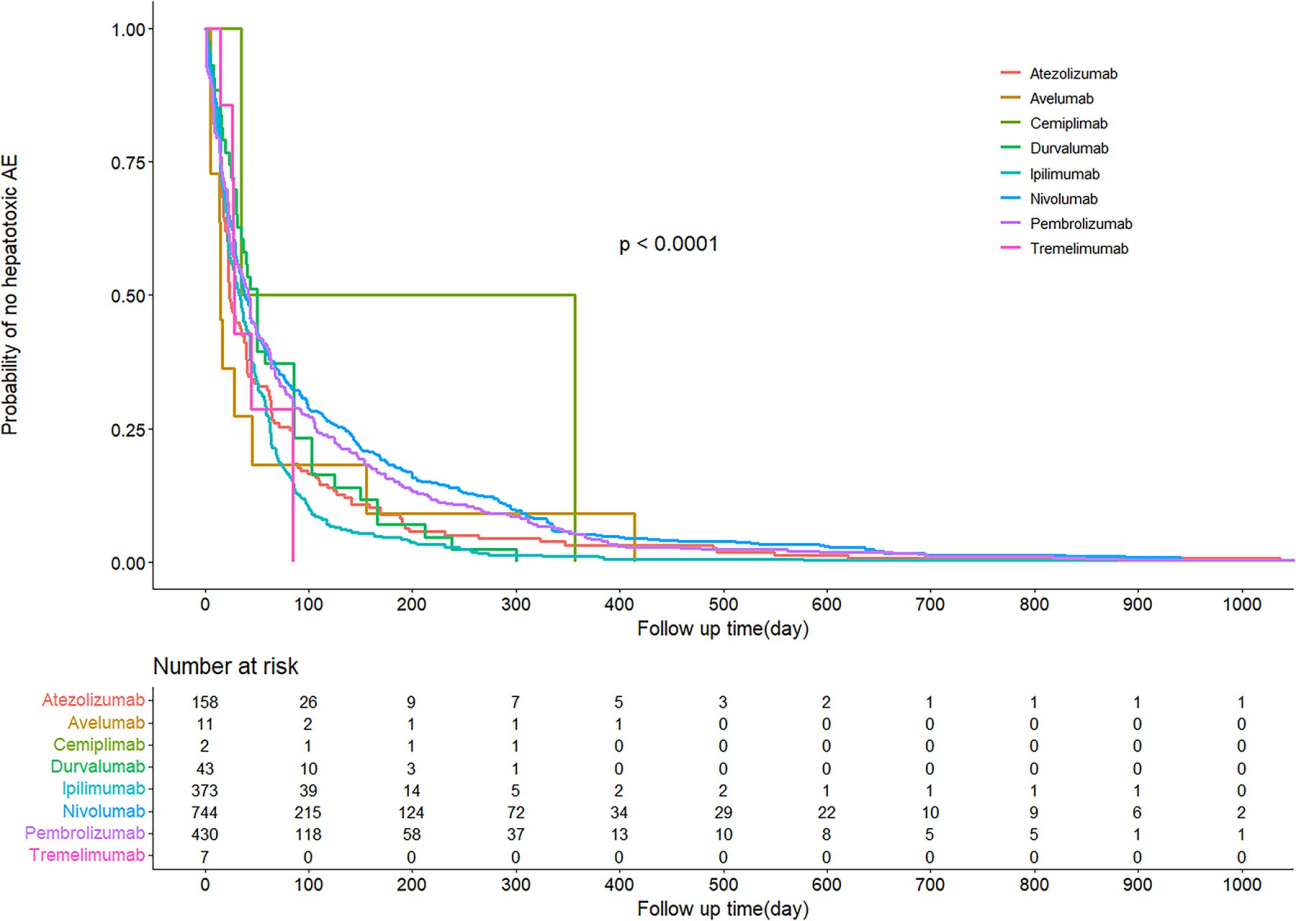
Kaplan-Meier curves and risk tables for AE reports of ICI-related hepatotoxicity.

A total of 1,768 ICI-associated hepatotoxic AEs were reported at time of onset. The Kaplan-Meier curves of AE onset time for different ICIs are shown in Fig 3, with a P-value less than 0.0001 after Kruskal-Wallis test, suggesting a significant difference in AE onset time for different ICIs. The risk table in the lower part of Fig 3 demonstrates the number of people followed up at each time point.

The median time to onset of action was 42 days (interquartile range (IQR) 16–98 days). Table 6 shows the time to onset of hepatotoxic AEs for each ICIs. The shortest median time to AE onset was 15 days for Avelumab (IQR 9.5–36.5) and 196 days for Cemiplimab (IQR 115–276.5).

**Table 6:** Time interval between episodes of hepatotoxic AEs for ICIs.

	Median time(day)	First quartile time(day)	Third quartile time(day)	IQR(day)
Atezolizumab	23.00	16.00	79.25	63.25
Avelumab	15.00	9.50	36.50	27.00
Cemiplimab	196.00	115.50	276.50	161.00
Durvalumab	50.00	24.00	86.00	62.00
Ipilimumab	33.00	15.00	63.00	48.00
Nivolumab	39.00	15.00	134.00	119.00
Pembrolizumab	42.00	15.00	106.75	91.75
Tremelimumab	28.00	26.50	64.50	38.00

**Fig 4:**
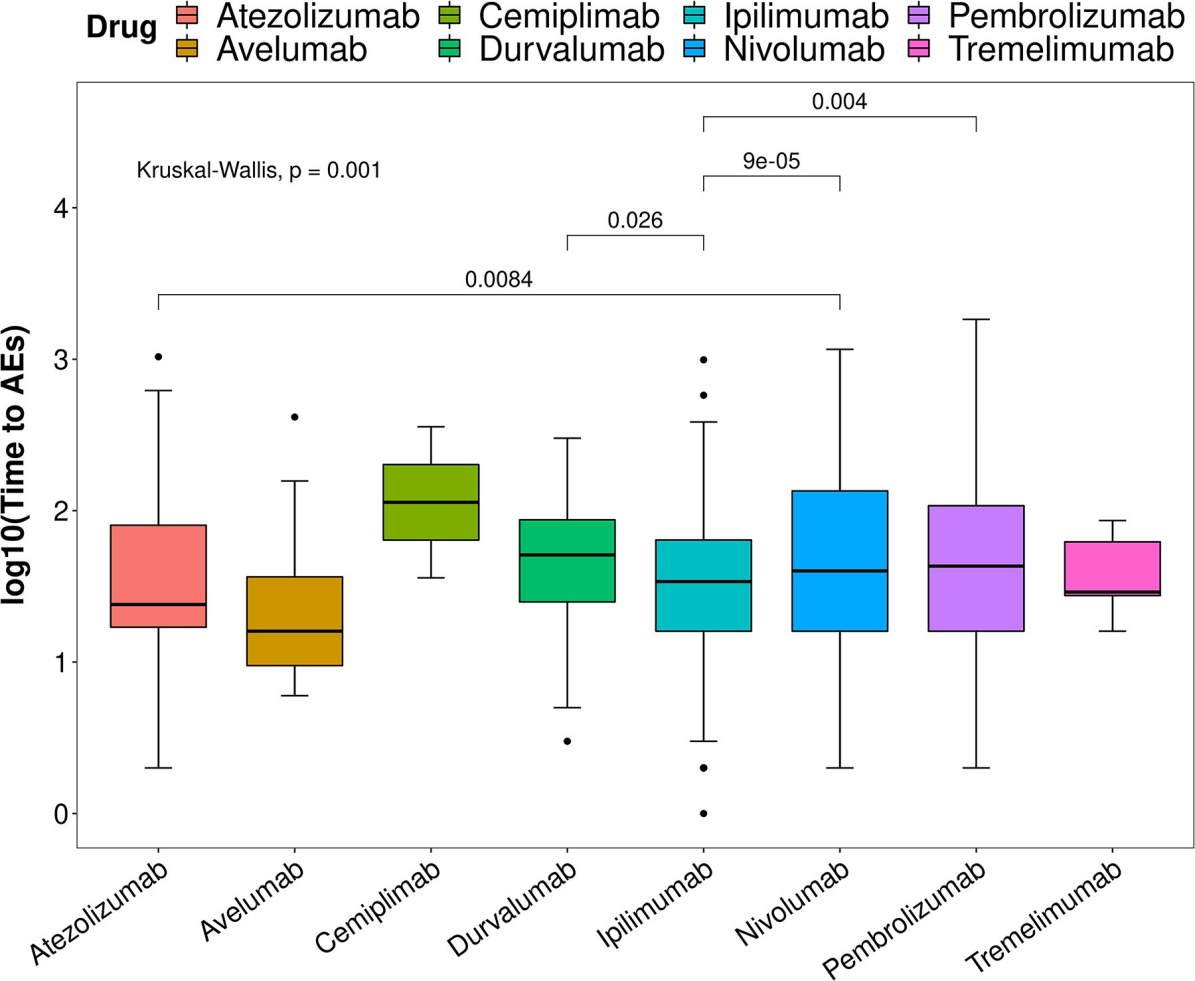
Analysis of differences in the time interval between episodes of hepatotoxic AEs for ICIs.

A two-by-two comparison of the different drugs by Wilcoxon rank sum test revealed that the time interval to AEs onset was significantly shorter for Ipilimumab than Durvalumab, Nivolumab and Pembrolizumab; Atezolizumab had a significantly shorter time to AEs onset than Nivolumab (Fig 4). Of these, the number of cases for Avelumab (11 cases), Cemiplimab (2 cases) and Tremelimumab (7 cases) was too small to be compared.

## 4. Discussion

Our study is the most systematic and comprehensive to date, based on the FAERS pharmacovigilance database, for comparing the association between ICIs and hepatic adverse reactions. We analyzed the FAERS database for adverse events associated with ICIs by measuring disproportionality and identified characteristics and differences between ICIs and associated hepatotoxic AEs in order to update safety information. We used two methods, the Bayesian Confidence Neural Network method (BCPNN) [15] and the Reported Odds Ratio method (ROR) [16]. The ICIs included in the study had varying dates of launch, with ipilimumab being the earliest to be launched in 2011. However, based on clinical use, nivolumab was the most used and widely available. Our study is a pharmacovigilance study based on more than 50 million records of ICI-related hepatotoxicity over a specific time period, which makes our conclusions more reliable.

Most clinical trials of ICIs have evaluated clinical effects other than AEs, and only brief descriptions of these severe or even fatal AEs have been provided. Previous studies have shown that ICIs increase the risk of organ system toxicity, such as endocrine, hematological, otologic and vagotoxicity [3, 17, 18]. Hepatotoxicity has also been mentioned in the adverse effects of some drugs. The delayed onset and prolonged duration of ICI-associated AEs compared with chemotherapy-induced AEs make timely recognition and early personalized management important [19].

Our results show that most ICIs-associated hepatotoxic AEs occur in the first few weeks after dosing, and then the likelihood decreases. However, it is still possible for patients to develop AEs during subsequent treatment, even over months or years [20]. Based on the analysis of the time interval of AE onset, the median time to AE onset was similar for ipilimumab, nivolumab and pembrolizumab, with the same incidence of AE on day 15 after dosing, but from the long-term follow-up, it was found that the incidence of AE on day 63 after dosing reached 75% for Ipilimumab, while pembrolizumab took 106.75 days and nivolumab took the longest of 134 days. This indicates that nivolumab has a lower risk of AE occurrence from long-term use, which may be due to the long duration of time nivolumab has been on the market and the clearer indications and adverse effects. Notably, the detection of reported hepatotoxic adverse events in the elderly produced a significant signal (IC025 = 0.2617, ROR025 = 1.3984), although a smaller proportion of elderly (≥65) than non-elderly (<65) were reported (36.78% vs. 44.50%). The results suggest that the elderly are at high risk of developing hepatotoxic AEs following ICIs drug therapy. Previous studies have shown that increased levels of hepatotoxicity in elderly patients are associated with elevated levels of oxidative stress and inflammation in the liver [21].

In addition, hepatitis and immune-mediated hepatitis have the strongest adverse effect signals and the widest distribution. the severity of ICI-associated immune-mediated hepatitis (IMH) varied. The risk of ICIs-induced liver injury may be influenced by the specific checkpoint molecules targeted, the dose level of the ICIs, and the preexisting autoimmune qualities, chronic infection or tumor cells infiltrating the liver parenchyma. The impact of ICIs therapy may be influenced by the presence of specific checkpoint molecules, ICIs dose levels and pre-existing autoimmune qualities, chronic infection or tumor cells infiltrating liver parenchyma. When patients experience liver injury during ICIs therapy, the cause of the injury should be promptly assessed and steps taken to best manage adverse events [22]. In addition, autoimmune hepatitis is another major immune-related hepatotoxic event, with a signal found in seven drugs (except avelumab), of which Ipilimumab had the strongest signal (IC025 = 4.2346, ROR025 = 23.1078). Previous studies have found that the use of ipilimumab in patients with previous autoimmune disease is more likely to cause immune-related AEs [23].

Liver metastases were detected as a signal in four drugs (Atezolizumab, Durvalumab, Ipilimumab, Nivolumab), however, it is generally believed that liver metastases are not caused by ICIs, that liver metastases are associated with poor prognosis and that ICIs therapy is less effective in patients with liver metastases. One study reported a shorter median progression-free survival time (MPFS) and lower disease control rate (DCR) in patients with liver metastases in NSCLC patients treated with Nivolumab [24].One possible explanation for the poor prognosis of patients with liver metastases treated with ICI is that patients with liver metastases have a poorer ECOG score standard PS score compared to patients without liver metastases. Less than 10% of patients with adenocarcinoma have a single liver metastasis, suggesting that multiple metastases may have occurred at the time of diagnosis of liver metastases and may have contributed to the poorer score [25]. In addition, the tumor microenvironment plays an important role in liver metastases. One study showed that patients with liver metastases showed lower CD8 T-cell counts at the invasive margin [26]. Considering that the liver has immunomodulatory functions to maintain local and systemic immune tolerance to auto- and foreign antigens, the association between liver metastases and CD8 T cells suggests that liver-induced peripheral tolerance may influence treatment outcome.

The mechanisms by which ICIs cause hepatotoxicity are currently being described differently. The hemi-antigen hypothesis mentions that reactive metabolites bind to cellular proteins to form neoantigens, called "haptens", which then move to major histocompatibility complex molecules on antigen-presenting cells and activate cytotoxic T lymphocytes, B cells and natural killer cells, stimulating an immune response against hepatocytes. The haptens may also induce autoantibodies to cytochrome p450 enzymes, leading to cellular damage and death [27, 28]. In addition, a more plausible explanation for the non-specific activation of the immune system associated with ICIs may lead to side effects in many organs, where in addition to attacking tumor-specific antigens, highly activated T lymphocytes also have targeting activity against normal tissues [29]. CD8+ cytotoxic T lymphocytes destroy tumor cells, causing them to release tumor antigens, neoantigens and autoantigens, leading to immune tolerance decline, which is referred to as epitope propagation [30]. By giving PD-1 immune tolerance-deficient mice amodiaquine, an antimalarial drug associated with drug-induced liver injury, Metushi et al. compared with immune-tolerant mice and found greater elevations in ALT and hepatic monocyte infiltration, suggesting that the severity of liver injury may be associated with reduced immune tolerance [31]. ZEN et al. also found in their study, liver biopsy samples from patients with ICI-associated hepatotoxicity showed predominantly lobular hepatitis and a significant infiltration of CD3+ and CD8+ T cells in the liver parenchyma of the samples. All the above studies support the mechanism of autoimmune attack of the liver leading to injury [32].

There are some certain limitations remain in our study. First, although safety issues can be assessed through the FAERS database, the database is limited by the lack of detailed clinical data. Further study needs carry out to construct the relationship between safety issue findings and practical clinical scenario. Moreover, due to lack of follow-up/censoring data, it is difficult to determine the causal relationship between ICI and adverse events in the hepatobiliary system. Finally, we compared ICIs with non-ICIs in the FAERS database using the proportional imbalance method in this article. In the future, we will compare AE signals with non-ICI drugs to enhance this research.

## 5. Conclusion

This study comprehensively assessed the association of ICIs with hepatotoxicity in real-world practice. Overall, a significant association was detected between ICIs and liver AEs and a relatively strong signal was detected in several ICIs immunotherapy regimens. Some of the results were consistent with previous literature. Immune-mediated hepatitis was associated with each drug and, in addition, autoimmune hepatitis was associated with most drugs (except Avelumab). In the clinical use of ICIs, patients should be alerted to the development of hepatotoxic AEs, especially in elderly patients. Clinicians need to be alerted to AEs associated with hepatotoxicity. The delayed onset and prolonged duration of AEs associated with ICIs makes timely recognition and early individualized management important.

## Supporting information

S1 Table (TIF)
