# Supplementary figures and images for: Hepatotoxicity in immune checkpoint inhibitors: A pharmacovigilance study from 2014–2021
Source: PLoS One. 2023 Mar 7;18(3):e0281983. doi: 10.1371/journal.pone.0281983 (PMC9990950; doi:10.1371/journal.pone.0281983)

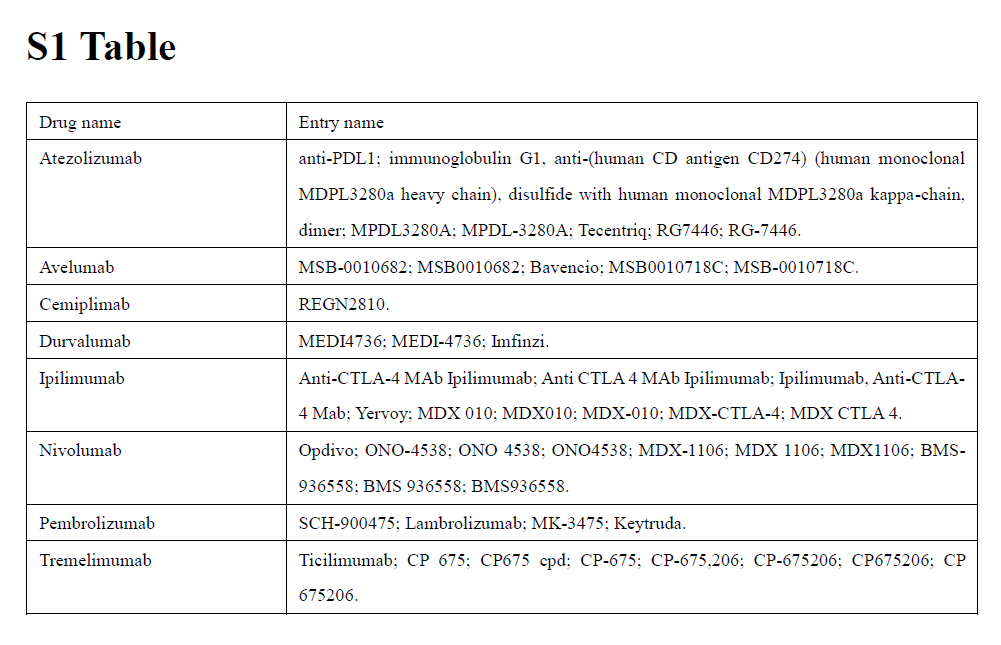

Supplement: S1 Table — (TIF) [file pone.0281983.s001.tif]
